# Plant-based diets and cardiovascular risk factors: a comparison of flexitarians, vegans and omnivores in a cross-sectional study

**DOI:** 10.1186/s40795-024-00839-9

**Published:** 2024-02-12

**Authors:** Anja Bruns, Theresa Greupner, Josefine Nebl, Andreas Hahn

**Affiliations:** Institute of Food Science and Human Nutrition, Leibniz University Hannover, Hannover, 30159 Germany

**Keywords:** Flexitarians, Vegans, Cardiovascular disease risk factors, CVD, Cholesterol, LDL, Pulse wave velocity, Metabolic syndrom severity score, HEI-Flex, Plant-based diet

## Abstract

**Background:**

The growing trend towards conscious and sustainable dietary choices has led to increased adoption of flexitarian diets, characterised by plant-based eating habits with occasional consumption of meat and processed meat products. However, the cardiovascular disease (CVD) risk factors associated with flexitarian diets compared to both vegans and omnivores remain underexplored.

**Methods:**

In this cross-sectional study, 94 healthy participants aged 25–45 years, categorized into long-term flexitarians (FXs ≤ 50 g/day of meat and meat products, *n* = 32), vegans (Vs, no animal products, *n* = 33), and omnivores (OMNs ≥ 170 g/day of meat and meat products, *n* = 29) were included. Various CVD risk factors were measured, including fasting blood samples for metabolic biomarkers, body composition analysis via bioimpedance, blood pressure measurements, arterial stiffness evaluated through pulse wave velocity (PWV) and metabolic syndrome (MetS) severity was determined using browser-based calculations (MetS-scores). Dietary intake was assessed using a Food Frequency Questionnaire (FFQ), diet quality was calculated with the Healthy Eating Index-flexible (HEI-Flex), while physical activity levels were recorded using the validated Freiburger questionnaire.

**Results:**

The data showed that FXs and Vs had more beneficial levels of insulin, triglycerides, total cholesterol, and LDL cholesterol compared to OMNs. Notably, FXs revealed the most favorable MetS-score results based on both BMI and waistline, and better PWV values than Vs and OMNs. In addition, FXs and Vs reported higher intake rates of vegetables, fruit, nuts/seeds and plant-based milk alternatives.

**Conclusion:**

The flexitarian diet appears to confer cardiovascular benefits. While Vs had the most favorable results overall, this study supports that reducing meat and processed meat products intake, as in flexitarianism, may contribute to CVD risk factor advantages.

**Supplementary Information:**

The online version contains supplementary material available at 10.1186/s40795-024-00839-9.

## Introduction

Plant-based diets have gained popularity in Germany and Western countries in general which is likely driven by increased awareness of sustainable lifestyles, animal welfare and health concerns [1, 2]. In addition, the flexitarian diet, which is plant-based but includes small amounts of meat and processed meat products, is winning followers who cite health aspects as their primary motivation [3, 4].

In recent years, cardiovascular disease (CVD) has remained the leading cause of death worldwide as well as in Germany, and more than half of all deaths are directly related to it [5, 6]. Consequently, when assessing the health effects of different dietary patterns, risk factors of CVD should be taken into account. However, the causes of CVD are diverse and can be divided into modifiable and non-modifiable risk factors. Non-modifiable risk factors include age, gender and genetic predisposition. In contrast, diet and lifestyle are important modifiable risk factors [1, 6–11].

Typical omnivore diets rich in meat and especially processed meat products have been shown to be associated with a higher prevalence of CVD risk factors such as obesity [12, 13], hypertension [14, 15], insulin resistance [7, 11], unfavourable blood lipid levels [10, 12] and adverse vascular changes [5, 7]. In Germany, dietary habits of omnivores are characterised by a high consumption of meat and processed meat products above the recommended intake rates (> 600 g/week) of the German Nutrition Society [16, 17]. Additionally, a physically active lifestyle (> 2.5 h/week of moderate activity) reduces the risk of development and progression of atherosclerosis, which is an important target for intervening and preventing CVD risk factors [18–20]. However, the physical activity levels are too low in western industrialized countries (< 2.5 h/week) [21], including Germany, with only 38% of people reaching the recommended activity rates [22].

While the multiple cardiovascular health benefits of an exclusively plant-based vegan diet have been widely described [23–28], current studies focusing on a plant-based flexitarian diet are still rare [29–32]. Therefore, it is unclear whether a diet that is healthy for the cardiovascular system necessarily excludes animal products, or whether a reduction in meat and processed meat products is sufficient to benefit from the health-promoting effects.

Although CVD usually occurs in older age, dietary and lifestyle factors in younger years play a crucial role in the development of the disease [33, 34]. Unfortunately, there is limited research on the CVD risk profile of FXs compared to Vs and OMNs in Germany. Thus, the aim of this cross-sectional study was to evaluate associations of a flexitarian diet compared to a vegan and omnivore diet on CVD risk factors in a young to middle-aged, healthy German study population.

This study was part of the interdisciplinary research project ‘NES’ (Nachhaltige Ernährungsstile) between the Leibniz University of Hannover and the Georg August University of Göttingen, Germany.

## Materials and methods

### Study design and participants

This cross-sectional study was conducted at the Institute of Food Science and Human Nutrition, Leibniz University of Hannover, Germany. Ethical approval was provided by the Ethics Commission of the Medical Chamber of Lower Saxony (Hannover, Germany) at 9th of September 2019 under 43/2019. The study was carried out between March and August 2020. However, the investigations only took place during the non-lockdown periods and only people who had not previously been infected with COVID were included in the study. Written informed consent was obtained from all participants in accordance to the guidelines of the Declaration of Helsinki. The study was registered in the German Clinical Trials Registry in January 2020 (DRKS 00019887).

The detailed study design has recently been published by Bruns et al., 2022 [35].

Interested subjects were included in the study if they followed their diet for at least ≥ 1 year and were categorized as follows: (a) flexitarians (FXs): meat and processed meat products consumption ≤ 50 g/day (equivalent to ≤ 350 g/week), (b) vegans (Vs): complete exclusion of food of animal origin, (c) omnivores (OMNs): meat and processed meat products consumption ≥ 170 g/day (equivalent to ≥ 1190 g/week). Meat and processed meat products were defined as red and white meat for meat and ham, sausage, cold cuts, meatballs and meat nuggets for meat products. Consumption limits for FXs were derived from national and international meat and processed meat products intake recommendations [17, 36, 37], and for OMNs on per capita consumption between 2011 and 2018 in Germany and Europe, respectively [38, 39]. Notably, to ensure a clear distinction between FXs and OMNs, subjects with a daily consumption of meat and processed meat products ≥ 50 g and ≤ 170 g were excluded.

Participant eligibility was assessed through a multi-step process. First, interested subjects were preselected via an online questionnaire, which mainly contained questions about the inclusion and exclusion criteria (e.g. age, sex, anthropometrics, health status, dietary habits) to check whether they were suitable for the FX, V or the OMN group. Secondly, potentially eligible participants subsequently underwent a face-to-face interview, which focused on dietary habits (e.g. the quantity of meat and processed meat products consumption) as well as lifestyle factors. Thirdly, only subjects who reported no change in their behaviour due to the pandemic situations were included in the study. Finally, as a result, the decision to participate in the study was made.

**Fig. 1:**
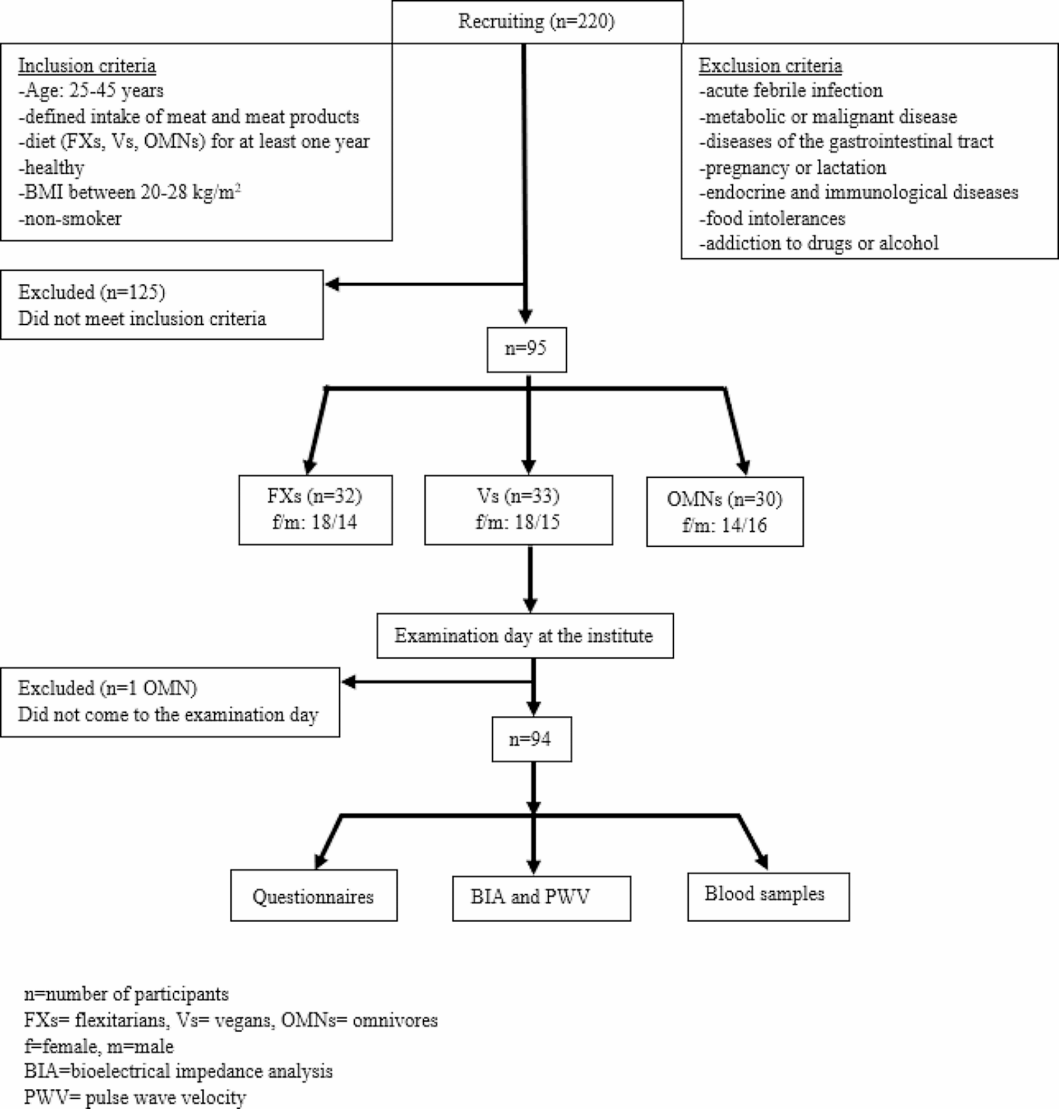
Flow chart of the study

Moreover, the study aimed to ensure a homogeneous cohort in terms of a narrow age range, gender, BMI within the physiological range (20 and 28 kg/m²) and non-smoker. The main exclusion criteria were: acute febrile infections, metabolic or malignant diseases, gastrointestinal disorders, pregnancy or lactation, endocrine and immunological diseases, food intolerances, and drug or alcohol dependence (Fig. 1). Finally, matched participants were invited to come to the Institute for an examination day.

### Anthropometric data and body composition

On the examination day, the participants’ height and weight were measured to calculate the BMI according to the standard formula [40]. Waist and hip circumferences were also determined using a tape measure. Body composition parameters were assessed by multi-frequency bioelectrical impedance analysis (BIA) according to the manufacturer’s guidelines using Nutriguard M (Data Input Company, Darmstadt, Germany).

### Food frequency, diet quality calculation and physical activity questionnaires

Dietary habits were recorded using the validated Food Frequency Questionnaire (FFQ) of the Robert Koch Institute (RKI), Berlin, Germany [41]. It consists of 57 questions with several sub-questions on dietary habits/food group intake in the previous 4 weeks. In addition, 28 questions were included on plant-based alternative products, low or highly processed, respectively.

Diet quality was calculated using the HEI-Flex score, which is a modification of the validated Healthy Eating Index-2015 (HEI-2015) [42]. Based on the FFQ data, a single HEI-Flex score value was calculated for each participant and then the median of each diet group was presented. In detail, information of diet quality calculations based on the HEI-Flex can be found elsewhere [35].

Health-relevant activity as a confounding factor (Appendix 1) was recorded using the validated German Freiburger questionnaire to assess the activity level of each participant [43].

### Arterial stiffness measurements

Arterial stiffness was determined by pulse wave velocity (PWV) analysis and blood pressure measurements. Both were taken according to the manufacturer’s recommendations of boso ABI-system 100 PWV, BOSCH + SOHN, Jungingen, Germany, 2019.

All measurements and analysis were carried out by trained nutritionists from the Institute.

### Biomarker analysis in blood and MetS-score calculation

After an overnight fast, the blood samples were obtained by an arm vein puncture and stored below 5 °C. Samples were transported to the accredited and certified Laboratory of Clinical Chemistry, Hannover Medical School, Germany, for analysis.

A photometric method (Beckman Coulter GmbH, Krefeld, Germany) was used for the analysis of fasting glucose, triglycerides, low-density lipoprotein (LDL) and high-density lipoprotein (HDL) cholesterol. High-pressure liquid chromatography (Bio-Rad Laboratories GmbH, Feldkirchen, Germany) was used to analyze HbA1c. Insulin concentrations were determined by electrochemiluminescence immunoassay (ECLIA) using cobas 801e (Roche Diagnostics GmbH, Mannheim, Germany).

For the assessment of insulin resistance, the Homeostatic Model Assessment (HOMA) was used according to the following formula: HOMA index = fasting insulin (µU/ml) x fasting blood glucose (mg/dl) / 405 [44].

The systemic immune inflammation index (SII) was estimated using the following formula: $SII = \frac{{PxN}}{L}$, where P, N and L are the numbers of peripheral platelets, neutrophils and lymphocytes, respectively [45].

The browser-based American Metabolic Syndrome (MetS) Severity Calculator was used to determine individual MetS severity using established calculations [46, 47]. These calculations take into account several CVD risk parameters, such as systolic blood pressure, triglycerides, HDL cholesterol and fasting glucose, as well as information on sex, age, race/ethnicity and weight. As a result, a single value is calculated for each person, usually using body mass index (MetS-score based on BMI). In addition, the MetS-score can also be calculated on the basis of waist circumference (MetS-score based on waist circumference). As there are advantages and disadvantages of using BMI and waist circumference to calculate the MetS-score, both methods were presented because (a) BMI correlates well with the percentage of total fat, but to a limited extent when the percentage of muscle mass in the total mass of an individual is high, and (b) waist circumference is a better predictor of metabolic risk than BMI. However, waist circumference is a less good measure of visceral fat in normal weight and younger subjects.

### Data analysis and statistical methods

**Fig. 2:**
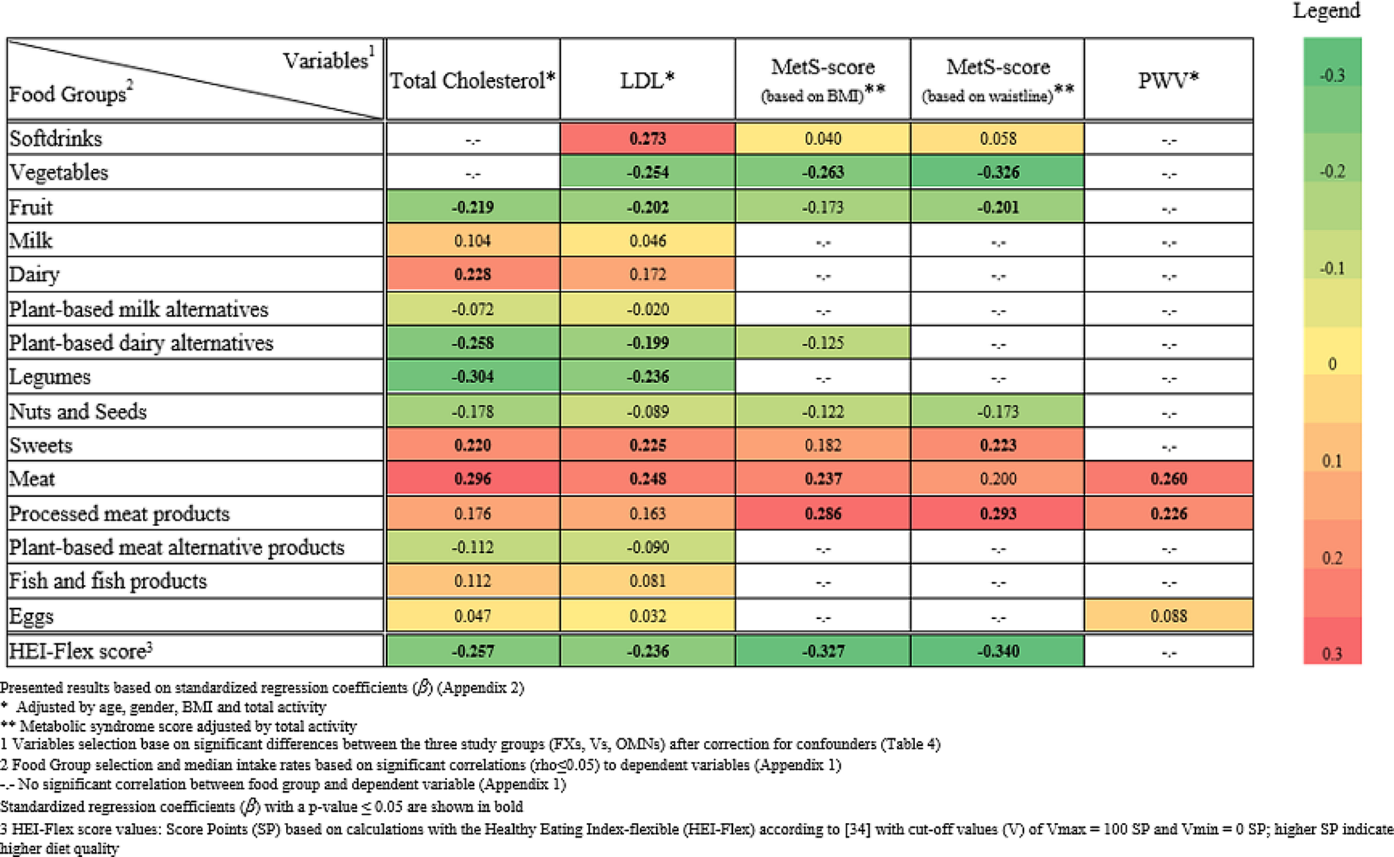
Heat map of standardized regression coefficients (β) obtained from linear regression analyses between median food group intakes and CVD risk parameters

Assuming an effect size ≥ 0.8, the sample size of *n* = 25 per group was based on a significance level (alpha) of 0.05 and a beta of 0.8 for detecting differences between the three diets. A minimum of 30 participants per group were enrolled, taking into account an expected drop-out rate of 15%. SPSS software (IBM SPSS Statistics Version 28.0.1.0; Chicago, IL, USA) was used for statistical analysis. Data are presented as median ($\tilde x$) and 25th-75th percentiles. The Kolmogorov-Smirnov test was used to test for normality. Normally distributed data were tested with univariate one-way analysis of variance (ANOVA) and Bonferroni correction for post hoc analysis to assess differences between the three dietary patterns. Non-normally distributed data were tested with the non-parametric Kruskal-Wallis test to detect statistically significant differences between the three groups. Regression calculations were performed stepwise: First, CVD risk factors were selected that differed significant between the three study groups after adjustment for confounders (total cholesterol, LDL, PWV and both MetS-sores). Second, to assess the relationship between these CVD risk factors and consumption amounts of food groups, the Spearmans rho correlation coefficient (rho) was used at the p_rho_≤0.05 level (Appendix 2) [48]. Third, linear regression models were applied to evaluate the associations between these CVD risk parameters and identified food groups intake (Appendix 3). To approach normality, all dependent variables were log-transformed. The residuals were tested for uniform linear dispersion. If homoscedasticity was present, a bootstrap was performed using the BCa-method. In the regression analysis, an adjusted association (age, sex, BMI and total activity) with the dependent variable (cholesterol, LDL, PWV) was included for each food group. Both MetS-score values were only adjusted for total activity, as age, sex and weight status were already taken into account in the scoring calculations. Heat-Map colours are based on adjusted standardized regression coefficients β (Fig. 2).

The statistically significance level of *p* ≤ 0.05 was used for all analyses.

## Results

### Anthropometric and body composition

Anthropometric and body composition measures indicate a healthy study collective (Table 1). Sex-specific values were only reported if there were statistically significant differences between the sex-specific diet groups. In all groups, the median BMI was within the normal range. Lower BMI values were observed for the FX women compared to the OMN women (*p* = 0.05), but body weight did not differ significantly between the three diet groups. Only for body fat were significant differences found. FX women had significantly lower values for both body fat in kg and in percent compared to OMN women (*p* = 0.013 and *p* = 0.003, respectively), whereas the difference was not significant for men. In addition, the V women had a significantly lower percentage of body fat compared to the OMN women.

**Table 1:** Anthropometric and body composition

Parameter		FXs	Vs	OMNs	*p*-value overall
Total participants, *n* (f/m)		32 (18/14)	33 (18/15)	29 (13/16)	0.641
Age (years)		32 (26–36)	33 (29–37)	32 (28–43)	0.377
**Anthropometry**					
Body weight (kg)		67.6 (63.7–79.6)	68.9 (64.0–80.0)	78 (71.2–80.6)	0.059
Body height (m)		1.76 (1.72–1.82)	1.73 (1.67–1.81)	1.76 (1.69–1.81)	0.523
Body-Mass-Index (kg/m^2^)	total	22.0 (21.0–25.0) *	23.0 (22.0–25.0)	25.0 (23.0–27.0)	**0.005**
	f	22.0 (21.0–23.0) *	23.0 (22.0–25.0)	26.0 (22.0–28.0)	**0.050**
	m	23.0 (21.0–25.0)	24.0 (22.0–25.0)	25.0 (23.0–26.0)	0.277
Waist circumference (cm)	total	74.0 (71.0–83.0)	78.0 (72.0–82.0)	78.0 (76.0–87.0)	0.257
	f	72.0 (70.0–76.0)	74.0 (68.0–78.0)	76.0 (68.0–85.0)	0.558
	m	79.0 (73.0–87.0)	82.0 (78.0–91.0)	79.0 (78.0–89.0)	0.449
**Body composition**					
Basal metabolic rate (kcal)		1380 (1330–1625)	1390 (1330–1580)	1510 (1350–1690)	0.404
Phase angle (°)		5.85 (5.50–6.30)	5.70 (5.40–6.10)	6.10 (5.60–6.60)	0.218
Body water (L)		35.3 (33.3–43.5)	36.0 (32.5–42.9)	41.6 (33.7–44.6)	0.704
Lean body mass (kg)		48.3 (45.5–59.5)	49.2 (44.4–58.7)	56.9 (46.0–61.0)	0.717
Body fat (kg)	total	18.8 (14.5–21.2) *	19.8 (16.6–22.7)	21.6 (17.8–27.0)	**0.040**
	f	19.0 (15.8–21.2) *	19.8 (18.6–21.6)	23.7 (20.5–31.7)	**0.013**
	m	16.9 (11.1–21.1)	18.5 (13.8–23.4)	19.3 (15.0-24.5)	0.378
Body fat (%)	total	25.6 (21.7–30.1)	28.5 (24.5–31.5)	28.3 (23.5–34.9)	0.156
	f	29.3 (25.8–33.0) *	30.8 (28.7–33.7) **	35.4 (32.5–40.7)	**0.003**
	m	1.7 (17.0-23.8)	25.2 (17.2–26.3)	23.9 (20.7–27.5)	0.225

FXs = flexitarians, Vs = vegans; OMNs = omnivores

n = number of participants

f = female; m = male

Data are shown as median ($\tilde x$) with 25th, 75th percentiles

Differences between groups were analyzed with One-way ANOVA for normally distributed data and Kruskal-Wallis test with post/hoc Bonferroni correction for non-normally distributed data

*p* < 0.05 was considered statistically significant

*p*-values in bold represent statistical significance

* statistically significant difference between FXs and OMNs

** statistically significant difference between Vs and OMNs

*** statistically significant difference between FXs and Vs

### Food group intake and diet quality between the three study groups

There were no significant differences in median consumption of beverages (low/no calorie), softdrinks (sugared) and bread/rice/noodles/potatoes between the three diet groups (Table 2). In contrast, OMNs consumed the least vegetables, FXs twice as much and Vs three times as much (*p* < 0.001), with significant differences between FXs and Vs compared to OMNs. Similarly, median fruit consumption was about twice as high in FXs and Vs compared to OMNs (*p* = 0.018), with only Vs and OMNs differing significantly.

Although FXs consumed in median only half as much milk as OMNs, the difference was not significant. The intake of plant-based milk alternatives was about five times lower for FXs than for Vs (*p* = 0.001), and also lower for plant-based dairy alternatives (*p* = 0.001). OMNs consumed in median neither plant-based milk nor plant-based dairy alternatives.

FXs and OMNs had significantly lower intake rates of legumes compared to Vs (*p* < 0.001). Regarding nuts/seeds, FXs reported an intake about 4 times higher than OMNs, but only about half as much as Vs (*p* < 0.001). The consumption of sweets and alcohol was not significantly different between FXs and Vs, but was significantly higher in OMNs than in Vs.

As expected, FXs had a significantly lower meat and processed meat products consumption than OMNs. The consumption of plant-based meat alternatives was highest among the Vs, significantly lower among the FXs and not consumed by OMNs (*p* < 0.001). The reported median intake of fish/fish products and eggs did not differ significantly between FXs and OMNs. However, OMNs consumed twice as many eggs as FXs.

The HEI-Flex score results differed significantly between all diet groups (*p* < 0.001) with Vs showing the most favorable diet quality, followed by FXs and then by OMNs.

**Table 2:** Food group intake and diet quality between the three study groups

Food group	FXs	Vs	OMNs	*p*-value overall
**Daily intake (g)**				
Beverages (low/free-caloric)	1950 (1463–3059)	2850 (1575–5110)	2391 (1639–3344)	0.136
Softdrinks (sugared)	7.00 (0.00–43.0)	18.0 (0.00–43.0)	43.0 (7.00-200)	0.091
Bread, Rice, Noodles, Potatoes	164 (93.8–275)	225 (176–317)	170 (137–267)	0.126
Vegetables	284 (142–396) *	375 (193–600)	107 (70.0-150) **	**< 0.001**
Fruit	254 (156–380)	308 (163–601)	141 (89.0-332) **	**0.018**
Milk	100 (21.4–200)	0.00 (0.00–0.00) ***	200 (42.8–200) **	**< 0.001**
Dairy	106 (42.2–199)	0.00 (0.00–0.00) ***	87.8 (52.6–204) **	**< 0.001**
Plant-based milk alternatives	17.9 (3.60–100) *	100 (21.4–400) ***	0.00 (0.00-3.60) **	**< 0.001**
Plant-based dairy alternatives	0.27 (0.00-14.2)	37.1 (21.8–50.8) ***	0.00 (0.00–0.00) **	**< 0.001**
Legumes	21.4 (6.70–34.8)	150 (75.0-150) ***	13.3 (5.36–32.1) **	**< 0.001**
Nuts and Seeds	12.0 (7.00-23.5) *	26.0 (14.0–52.0) ***	3.00 (2.00–13.0) **	**< 0.001**
Sweets	124 (76.5-188.5)	107 (75.0-142)	175 (87.0-234) **	**0.033**
Alcohol	3.50 (0.50–10.0)	1.00 (0.00–7.00)	8.00 (4.00–14.0) **	**0.009**
**Weekly intake (g)**				
Meat	75.0 (33.7–198) *	0.00 (0.00–0.00) ***	513 (405–645) **	**< 0.001**
Processed meat products	77.5 (42.1–168) *	0.00 (0.00–0.00) ***	502 (290–825) **	**< 0.001**
Plant-based meat alternative products	30.0 (0.00-180)	420 (180–490) ***	0.00 (0.00–0.00) **	**< 0.001**
Fish and fish products	56.2 (22.5–112)	0.00 (0.00–0.00) ***	78.7 (28.1–157) **	**< 0.001**
Eggs	90.0 (37.5–195)	0.00 (0.00–0.00) ***	180 (75.0-210) **	**< 0.001**
**Dietary quality (score points)**				
HEI-Flex score^1^	54 (49–63) *	61 (54–70) ***	47 (43–55) **	**< 0.001**

FXs = flexitarians, Vs = vegans; OMNs = omnivores

Data are shown as median ($\tilde x$) with 25th, 75th percentiles

Difference between groups were analyzed using either Kruskal Wallis with Post/hoc Bonferroni correction

*p* < 0.05 was considered statistically significant

*p*-values in bold represent statistical significance

* statistically significant difference between FXs and OMNs

** statistically significant difference between Vs and OMNs

*** statistically significant difference between FXs and Vs

^**1**^HEI-Flex score values: Score points (SP) based on calculations with the Healthy Eating Index-flexible according to [35] with cut-off values (V) of: Vmax = 100 SP and Vmin = 0 SP; higher SP indicate higher diet quality

### Comparison of CVD risk profile parameters between the three study groups

The median values of CVD risk markers were within the reference ranges in all diet groups (Table 3).

Observing of **blood glucose markers** in the three study groups showed similar levels of fasting glucose, HbA1c and HOMA, while Vs had the lowest fasting insulin concentrations compared to FXs and OMNs (*p* = 0.016), with statistical significance between Vs and OMNs.

Regarding **blood lipid markers**, both FXs and Vs had significantly lower levels than OMNs for total and LDL-cholesterol (*p* < 0.001). HDL-cholesterol levels were not statistically different between the three groups. Moreover, FXs and Vs had significantly lower fasting triglycerides than OMNs (*p* = 0.008).

Concerning the **inflammatory state**, no significant difference was observed between the three diet groups in the SII.

In terms of **metabolic syndrome** (MetS) severity, FXs had lower (more favorable) score levels, closely followed by Vs and significantly better than OMNs based on BMI (*p* = 0.012) and waist circumference (*p* = 0.027). However, all diet groups had MetS-score values associated with a low risk (MetS-score < 0) of CVD events [47].

Regarding **vascular health parameters**, there were no significant differences between the three diet groups for systolic and diastolic blood pressure. Notably, significantly lower (better) values (*p* = 0.022) were observed for PWV in the flexitarian group compared to Vs and OMNs.

Further examination of whether the associations on CVD risk factors remained significant after adjustment for covariates showed that there were still significant differences between the three dietary groups for total cholesterol, LDL, both MetS-scores and PWV levels. However, the differences in triglyceride concentrations and insulin lost significance after correction for confounders (age, sex, BMI, total activity).

**Table 3:** Comparison of CVD risk profile parameters between the three study groups

Parameter	FXs	Vs	OMNs	*p*-value overall
**Blood glucose markers**				
Glucose (mmol/L)	5.00 (4.70–5.15)	5.00 (4.80–5.20)	5.00 (4.90–5.30)	0.600
HbA1_C_ (%)	5.10 (5.00-5.20)	5.00 (4.80–5.10)	5.10 (5.00-5.20)	0.175
Insulin (mU/L)	5.55 (3.90–8.65)	5.40 (4.20–6.20)	6.80 (5.60–9.80) **	**0.016**
HOMA	1.00 (0.71–1.32)	1.06 (0.73–1.35)	1.22 (0.94–2.06)	0.300
**Blood lipid markers**				
Cholesterol (mmol/L)	4.35 (4.00-5.05) *	3.80 (3.30–4.20) ***	4.90 (4.00-5.50) **	**< 0.001**
LDL-Cholesterol (mmol/L)	2.64 (2.11–3.47) *	2.14 (1.88–2.56) ***	3.17 (2.40–3.64) **	**< 0.001**
HDL-Cholesterol (mmol/L)	1.68 (1.48–1.88)	1.62 (1.23–1.81)	1.57 (1.29–1.72)	0.375
Triglycerides (mmol/L)	0.73 (0.61-1.00) *	0.77 (0.59–0.98)	1.14 (0.82–1.36) **	**0.008**
**Inflammatory state**				
SII	411 (271–581)	359 (306–477)	369 (292–474)	0.950
**Metabolic Syndrome (MetS) scores**				
MetS (based on BMI)	-1.00 (-1.24- -0.57) *	-0.87 (-1.05- -0.36)	-0.56 (-0.83- -0.06)	**0.012**
MetS (based on waistline)	-1.03 (-1.33- -0.61) *	-1.00 (-1.28- -0.55)	-0.60 (-0.99- -0.20)	**0.027**
**Parameters of vascular health**				
Systolic blood pressure (mmHg)	122 (114–132)	123 (119–130)	128 (124–137)	0.064
Diastolic blood pressure (mmHg)	74.0 (70.0–78.0)	76.0 (70.0–81.0)	79.0 (75.0–84.0)	0.082
Pulse wave velocity (m/sec)	5.60 (5.10–6.20) *	5.90 (5.10–6.10)	6.10 (5.70–6.70)	**0.022**

FXs = flexitarians, Vs = vegans; OMNs = omnivores

Data are shown as median (x ~) with 25th, 75th percentiles

Differences between groups were analyzed with One-way ANOVA for normally distributed data and Kruskal-Wallis test with post/hoc Bonferroni correction for non-normally distributed data

*p* < 0.05 was considered statistically significant

*p*-values in bold represent statistical significance

* statistically significant difference between FXs and OMNs

** statistically significant difference between Vs and OMNs

*** statistically significant difference between FXs and Vs

SII: Systemic-Immune-Inflammation Index

HOMA: Homeostasis Model Assessment Index according to [44]

MetS-score: Metabolic Syndrome Severity Score based on BMI resp. waistline according to [46, 47]

### Associations between CVD risk factors and food groups intake

In relation to total cholesterol, significant positive associations were observed for dairy products, sweets and meat consumption (Fig. 2, Appendix 3), with dairy and meat intake showing the most pronounced associations (β ≥ 0.220). Conversely, inverse significant relationships were found for intake of fruit, plant-based dairy alternatives, legumes and HEI-Flex score points, with standardized regression coefficients of β≤-0.219. No significant associations were observed for median intakes of plant-based meat and milk alternatives.

For LDL cholesterol, significant positive associations were found between median consumption of softdrinks, sweets and meat (β ≥ 0.225). Conversely, statistically significant negative associations emerged for median intake of vegetables, fruit, dairy alternatives, legumes and HEI-Flex score points (β≤-0.199).

For the two MetS-scores (based on BMI and waistline), significant positive associations were found with processed meat consumption (β ≥ 0.286). In addition, the MetS-score based on BMI exhibited significant associations with median meat consumption (β = 0.237), while the MetS-score based on waistline indicated a significant relationship with median sweets intake (β = 0.223). Conversely, significant negative coefficients were found between both MetS-scores and median vegetable intake as well as HEI-Flex scores (β≥-0.263). Likewise, a negative relationship was evident between MetS-score based on waistline and fruit intake (β≤-0.201). For PWV, significant positive associations were observed for the median consumption of meat and processed meat, respectively (β ≥ 0.226).

Overall, the regression analyses showed higher β-coefficients for animal-based food groups (β > 0), indicating adverse associations with CVD risk indicators. Conversely, higher median intakes of plant-based food groups were often corresponding to negative β-coefficients (β < 0), indicating a favorable association with the CVD risk profile.

## Discussion

Dietary choices play a crucial role in influencing the CVD risk [1, 49, 50]. While recent studies have already described cardiovascular health benefits for Vs and vegetarians [51], data on flexitarianism are still insufficient. As the consumption of meat and processed meat products is associated with an unfavorable CVD risk profile [52–54], the aim of the present study was to evaluate whether a cardiovascular-healthy diet requires the complete elimination of all animal products, as in veganism, or whether a reduced consumption of meat and processed meat products towards a more plant-based diet, as in flexitarianism, already supports beneficial outcomes on CVD risk factors. Therefore, a healthy, adult German study cohort with clearly defined FXs, Vs and OMNs was included.

The results of the present study were compared with data from different dietary patterns along the plant-based spectrum because, on the one hand, a precise and generally accepted definition of flexitarianism is still lacking [55, 56] and, on the other hand, previous research with clearly defined flexitarian study groups is rare. In addition, other studies often include self-defined dietary groups, had a higher proportion of women [31, 32, 57], a wider age range [9, 29, 32, 57], or participants with pre-existing conditions [27, 58, 59]. In contrast, the present study not only records the median consumption of various food groups, but also differentiates between processed foods and plant-based alternative products. Importantly, this study included both the “classic” individual blood parameters of CVD risk and also sum scores to assess the severity of MetS as an important CVD risk factor. Additionally, arterial stiffness was determined by measuring PVW as a CVD risk marker. Therefore, the present study results have an exploratory pilot character and data were not adjusted for multiple testing [60].

As is already known from studies comparing the beneficial CVD impact of a vegetarian versus an OMN diet, the present results supports these findings also for the flexitarian dietary pattern. The FXs (and Vs) showed a more favorable CVD risk profile in terms of blood lipid profile (total cholesterol, LDL), MetS-scores and PWV compared to OMNs. However, there were no statistically significant differences between the three study groups in blood glucose markers, blood pressure levels and inflammatory state. The reasons for this are unclear and may be due to the fact that the participants were young and healthy. In addition, there were also no significant differences in total energy intake between the groups, as recently published [61].

### Blood lipid profile parameters

The results supported that a higher consumption of vegetables, fruit, and legumes was associated with lower **total cholesterol and LDL** levels. Similarly, both FXs and Vs had significantly lower concentrations of total and LDL cholesterol compared to OMNs. These findings are consistent with a recent study (*n* = 258) which observed significantly lower levels of total and LDL cholesterol in several plant-based diets, including FXs, compared to OMNs [31]. Likewise, other studies have reported lower values of total and LDL cholesterol in non-OMN participants [10, 62–64].

**Triglyceride** concentrations in the current study were also significantly higher in OMNs than in FXs and Vs, but the difference lost significance after adjustment for confounders (age, sex, BMI, physical activity). However, previous research has also shown conflicting results regarding the effect of a plant-based diet compared to an omnivore diet on triglyceride levels. While a meta-analysis (2018) observed significantly lower triglyceride values in Vs compared to OMNs [65], another (2015) found no significant difference between a vegetarian diet compared to an omnivore diet [66]. Moreover, there were no significant differences in **HDL cholesterol** levels between the two plant-based diets (FXs and Vs) and OMNs in the present study. These results are consistent with other studies that have also found no differences in HDL levels between various plant-based diets and OMNs [31, 67].

### MetS-score calculations

In the present study, both FXs and Vs had lower (better) **MetS-score** levels compared to OMNs. In particular, FXs appeared to have the most beneficial values of the three diet groups. Notably, all groups achieved results associated with an intermediate (MetS-score = 0) or low (MetS-score < 0) risk level [46, 47]. These findings are in line with a cross-sectional analysis of the Adventist Health Study 2 (*n* = 773), which also found a lower risk of MetS in semi-vegetarians compared to OMNs [68]. Also, a more recent review (2021) showed, that a vegan diet seems to be useful in the prevention and treatment of MetS [69]. However, in the absence of comparable European or German results, the present values are compared with the U.S. population sample, which may limit direct comparability due to potential national differences, e.g. dietary habits. Nonetheless, the utilization of the MetS-scores is promising as it avoids relying on fixed cut-off values that are traditionally used for estimating the metabolic syndrome risk and enabling the identification of individuals with scores below a threshold who would not typically be considered at risk for CVD.

### Pulse wave velocity levels

Notably, FXs had significantly lower (more favorable) **pulse wave velocity** compared to OMNs in this study, even after adjusting for confounding factors. These findings are consistent with a study by Acosta-Navarro and colleagues (*n* = 88), who examined PWV in healthy male vegetarians and OMNs (age ≥ 35 years) and found significantly lower levels for vegetarians compared to OMNs [70]. Other studies have also reported improved vascular structure in participants following a plant-based diet compared to OMNs [10, 71, 72].

### Associations between food group intake, diet quality and CVD risk parameters

The results of the present study suggest that higher median consumption rates of softdrinks, dairy products, sweets, meat and processed meat are associated with higher total and LDL cholesterol levels, MetS-scores and PWV values. Additionally, it was observed that both FXs and Vs had significantly lower intake rates of sweets compared to OMNs. These findings are consistent with a cohort study (*n* = 17,824), which also reported higher consumption of sugary foods like softdrinks and sweets among OMNs compared to vegetarians [73]. Similarly, a recent review from 2020 highlighted that increased consumption of sweets, typically rich in free sugars and refined starches, is associated with a higher risk of obesity and elevated LDL levels [74].

As defined, OMNs had significant higher median consumption of meat and processed meat products compared to FXs and Vs in this study. Further, positive associations were observed between meat and particularly processed meat consumption and total and LDL cholesterol levels, MetS-scores, and PWV values. These findings are consistent with previous studies that have related meat and processed meat consumption to various CVD risk factors [9, 75–77]. For example, a cohort study (*n* = 81,529) concluded, that an increased red meat consumption was associated with higher cholesterol levels, hypertension and higher body weight [54], and a systemic review and meta-analysis of cohort studies (2019) found evidence, that a reduction in processed red meat intake is associated with a lower risk for CVD [78].

Furthermore, both FXs and Vs reported a high median consumption of plant-based milk, plant-based dairy, and meat alternatives, while OMNs reported no consumption of these products. However, the associations between these food groups and CVD risk factors were inconclusive in the present study. However, the impact of these products on human health are still not well investigated, as they vary greatly in composition and many are highly processed, containing high levels of salt, sugar, and/or saturated fat [79, 80]. These ingredients have been associated to a higher CVD risk, but their overall effects are still debated [81–83].

In terms of **diet quality**, based on the HEI-Flex score values, both FXs and Vs had significantly higher, more favorable, levels compared to OMNs. Furthermore, the regression calculations in the present study supported that higher score points (= higher diet quality) were associated with a more favorable blood lipid profile as well as MetS-scores. These findings are in line with previous studies that found inverse associations between diet quality and blood lipid parameters [84, 85]. Additionally, a cross-sectional polish study (*n* = 535) reported an inverse relationship between HEI-2015 scores and the occurrence of the metabolic syndrome [86].

### Strengths and limitations

The study has several limitations that should be considered. Firstly, the cross-sectional design limits the ability to establish causality between dietary patterns and CVD risk factors. Future research employing longitudinal or intervention designs would provide more robust evidence. Secondly, the sample size of 94 participants does not allow the generalizability of the findings. Therefore, it is important to interpret the results as findings from an exploratory pilot study. The latter, the use of food frequency questionnaires to assess dietary intake, may lead to recall bias and inaccuracies in reporting. Also, the dietary assessment method did not allow the accurate capture of dietary fiber intake, different fatty acids (MUFAs, PUFAs), and phytochemicals.

Despite these limitations, the study has several strengths. The well-controlled study design ensured homogeneity of the three dietary groups in terms of age, sex, BMI, health and smoking status. Notably, in contrast to previous studies, the present study also included MetS-scores and PWV as additional CVD risk indicators.

## Conclusion

In conclusion, both plant-based diets, FXs and Vs, were associated with improved blood lipid profiles and higher diet quality compared with OMNs in the present study. FXs were often intermediate between Vs and OMNs, with some CVD risk parameters approaching or exceeding those of Vs. Notably, FXs had the most favorable MetS-scores and PWV values compared to the other two groups. Overall, the results supported a beneficial impact of a flexitarian diet on CVD risk parameters in the present cohort. However, further research with larger, clearly defined flexitarian study populations is needed to better understand the influence of this dietary pattern on CVD risk factors.

### Electronic supplementary material

Below is the link to the electronic supplementary material.

Supplementary Material 1: Appendix 1: Health-relevant activity levels

Supplementary Material 2: Appendix 2: Indications of correlations between different food groups and CVD risk parameters

Supplementary Material 3: Appendix 3: A Linear regression models to examine associations of total cholesterol levels with various food groups in the total study population B Linear regression models to examine associations of LDL levels various food groups in the total study population. C Linear regression models to examine associations of MetS-score levels (based on BMI and waistline) with various food groups in the total study population. D Linear regression models to examine associations of PWV levels with various food groups in the total study population

## Data Availability

The datasets used and/or analyzed during the current study are available from the corresponding author on reasonable request.
