# Supplementary material for: Plant-based diets and cardiovascular risk factors: a comparison of flexitarians, vegans and omnivores in a cross-sectional study
Source: BMC Nutr. 2024 Feb 12;10:29. doi: 10.1186/s40795-024-00839-9 (PMC10860304; doi:10.1186/s40795-024-00839-9)
Supplement: Supplementary file 1 — Supplementary Material 1: Appendix 1: Health-relevant activity levels [file 40795_2024_839_MOESM1_ESM.docx]

**Appendix 1**

**Comparison of health-relevant activities between the three study groups**

| **Parameter** | **FXs** | **Vs** | **OMNs** | **p-value overall** |
| --- | --- | --- | --- | --- |
| Basic activity (h/week) | 3.50 (2.75-6.15) | 3.50 (1.70-7.00) | 2.00 (1.00-4.50) | 0.128 |
| Leisure activities (h/week) | 2.65 (1.00-5.00) | 2.00 (1.00-5.00) | 1.50 (0.80-3.50) | 0.294 |
| Sports activities (h/week) | 2.90 (1.45-4.50) | 5.00 (2.00-7.00) | 2.20 (0.50-3.00) ****** | **0.008** |
| Total activity (h/week) | 10.2 (7.70-14.7) * | 12.0 (7.50-16.1) | 7.80 (4.00-9.30) ****** | **0.004** |
| Night sleep (h/night) | 7.00 (7.00-7.50) | 7.00 (7.00-7.50) | 7.50 (7.00-8.00) | 0.108 |

FXs=flexitarians, Vs=vegans; OMNs=omnivores

h=hours

Data are shown as median ($\tilde{x}$) with 25^th^, 75^th^ percentiles

Difference between groups were analyzed using Kruskal Wallis with Post/hoc Bonferroni correction

p<0.05 was considered statistically significant

p-values in bold represent statistical significance

***** statistically significant difference between FXs and OMNs

****** statistically significant difference between Vs and OMNs

******* statistically significant difference between FXs and Vs
