# Supplementary material for: Plant-based diets and cardiovascular risk factors: a comparison of flexitarians, vegans and omnivores in a cross-sectional study
Source: BMC Nutr. 2024 Feb 12;10:29. doi: 10.1186/s40795-024-00839-9 (PMC10860304; doi:10.1186/s40795-024-00839-9)
Supplement: Supplementary file 2 — Supplementary Material 2: Appendix 2: Indications of correlations between different food groups and CVD risk parameters [file 40795_2024_839_MOESM2_ESM.docx]

**Appendix 2 Indications of correlations between different food groups and CVD risk parameters**

| **CVD risk parameters^1^** | **Cholesterol** | | **LDL Cholesterol** | | **MetS-score**  **(based on BMI)** | | **MetS-score**  **(based on waistline)** | | **Pulse wave velocity** | |
| --- | --- | --- | --- | --- | --- | --- | --- | --- | --- | --- |
| **Correlation results**  **Food Groups** | rho | p_rho_-value | rho | p_rho_-value | rho | p_rho_-value | rho | p_rho_-value | rho | p_rho_-value |
| Beverages (low/free-caloric) | 0.007 | 0.946 | 0.033 | 0.750 | -0.020 | 0.848 | -0.101 | 0.334 | -0.025 | 0.810 |
| Softdrinks (sugared) | 0.191 | 0.065 | 0.271** | 0.008 | 0.451** | 0.000 | 0.422** | 0.000 | 0.106 | 0.308 |
| Bread. Rice. Noodles. Potatoes | -0.158 | 0.129 | -0.141 | 0.175 | 0.140 | 0.180 | 0.156 | 0.132 | 0.129 | 0.214 |
| Vegetable | -0.195 | 0.060 | -0.280** | 0.006 | -0.316** | 0.002 | -0.335** | 0.001 | 0.029 | 0.785 |
| Fruit | -0.223* | 0.031 | -0.266** | 0.010 | -0.252* | 0.014 | -0.264** | 0.010 | 0.083 | 0.429 |
| Milk | 0.398** | 0.000 | 0.331** | 0.001 | 0.055 | 0.600 | 0.061 | 0.558 | 0.093 | 0.374 |
| Dairy | 0.422** | 0.000 | 0.346** | 0.001 | 0.033 | 0.749 | 0.032 | 0.757 | 0.072 | 0.491 |
| Plant-based milk alternatives | -0.229* | 0.026 | -0.247* | 0.016 | -0.121 | 0.245 | -0.129 | 0.214 | -0.153 | 0.140 |
| Plant-based dairy alternatives | -0.344** | 0.001 | -0.338** | 0.001 | -0.215* | 0.037 | -0.173 | 0.096 | -0.014 | 0.890 |
| Legumes | -0.359** | 0.000 | -0.354** | 0.000 | -0.127 | 0.222 | -0.129 | 0.214 | 0.034 | 0.743 |
| Nuts and Seeds | -0.217* | 0.036 | -0.218* | 0.034 | -0.241* | 0.019 | -0.258* | 0.012 | -0.046 | 0.661 |
| Sweets | 0.211* | 0.041 | 0.243* | 0.018 | 0.211* | 0.041 | 0.257* | 0.012 | 0.071 | 0.494 |
| Alcohol | 0.172 | 0.097 | 0.136 | 0.192 | 0.113 | 0.277 | 0.073 | 0.484 | 0.181 | 0.081 |
| Meat | 0.406** | 0.000 | 0.385** | 0.000 | 0.233* | 0.024 | 0.219* | 0.034 | 0.213* | 0.039 |
| Processed meat products | 0.358** | 0.000 | 0.367** | 0.000 | 0.246* | 0.017 | 0.256* | 0.013 | 0.282** | 0.006 |
| Plant-based meat alternative products | -0.325** | 0.001 | -0.293** | 0.004 | -0.079 | 0.447 | -0.105 | 0.316 | -0.192 | 0.063 |
| Fish and fish products | 0.386** | 0.000 | 0.348** | 0.001 | 0.002 | 0.982 | 0.063 | 0.546 | 0.189 | 0.069 |
| Eggs | 0.287** | 0.005 | 0.257* | 0.012 | -0.007 | 0.946 | 0.076 | 0.467 | 0.204* | 0.049 |
| HEI-Flex score^2^ | -0.267** | 0.009 | -0.296** | 0.004 | -0.354** | 0.000 | -0.349** | 0.001 | -0.008 | 0.938 |

1 selection bases on significant differences between the three study groups (FXs, Vs and OMNs) after correction for confounders

2 HEI-Flex score values: Score Points (SP) based on calculations with the Healthy Eating Index-flexible (HEI-Flex) according to [Bruns et al, 2022] with cut-off values [V] of: Vmax = 100 SP and Vmin = 0 SP; higher SP indicate higher diet quality

r_rho_=correlation coefficient according to Spearmans rho [J. Cohen, 1988]

** correlation is statistically significant at the 0.01 level (two-sided)

* correlation is statistically significant at the 0.05 level (two-sided)
