# Supplementary material for: Plant-based diets and cardiovascular risk factors: a comparison of flexitarians, vegans and omnivores in a cross-sectional study
Source: BMC Nutr. 2024 Feb 12;10:29. doi: 10.1186/s40795-024-00839-9 (PMC10860304; doi:10.1186/s40795-024-00839-9)
Supplement: Supplementary file 3 — Supplementary Material 3: Appendix 3: A Linear regression models to examine associations of total cholesterol levels with various food groups in the total study population B Linear regression models to examine associations of LDL levels various food groups in the total study population. C Linear regression models to examine associations of MetS-score levels (based on BMI and waistline) with various food groups in the total study population. D Linear regression models to examine associations of PWV levels with various food groups in the total study population [file 40795_2024_839_MOESM3_ESM.docx]

**Appendix 3**

**A Linear regression models to examine associations of total cholesterol levels with various food groups in the total study population**

| Total Cholesterol | Model 1* | | | Model 2** | | |
| --- | --- | --- | --- | --- | --- | --- |
|  | *R^2^* | p-value | *β* | *R^2^* | p-value | *β* |
| Food Groups^1^ |  | | | | | |
| Fruit | 0.040 | **0.029** | -0.225 | 0.213 | **0.028** | -0.225 |
| Milk | 0.005 | 0.230 | 0.125 | 0.180 | 0.273 | 0.104 |
| Dairy | 0.113 | **<0.001** | 0.349 | 0.218 | **0.022** | 0.228 |
| Plant-based milk alternatives | 0.010 | 0.725 | -0.037 | 0.174 | 0.475 | -0.072 |
| Plant-based dairy alternatives | 0.066 | **0.007** | -0.275 | 0.238 | **0.006** | -0.258 |
| Legumes | 0.098 | **0.001** | -0.328 | 0.253 | **0.002** | -0.304 |
| Nuts and Seeds | 0.030 | 0.053 | -0.200 | 0.198 | 0.081 | -0.178 |
| Sweets | 0.043 | **0.025** | 0.231 | 0.217 | **0.023** | 0.220 |
| Meat | 0.135 | **<0.001** | 0.380 | 0.249 | **0.003** | 0.296 |
| Processed meat products | 0.039 | **0.032** | 0.222 | 0.198 | 0.080 | 0.176 |
| Plant-based meat alternative products | 0.003 | 0.398 | -0.088 | 0.181 | 0.266 | -0.112 |
| Fish and fish products | 0.006 | 0.219 | 0.128 | 0.181 | 0.254 | 0.112 |
| Eggs | 0.005 | 0.483 | 0.073 | 0.171 | 0.623 | 0.047 |
| HEI-Flex^2^ | 0.076 | **0.004** | -0.293 | 0.230 | **0.010** | -0.257 |

1 Food Group selection base on significant Spearmans correlation coefficient rho ≤0.05 [J. Cohen, 1988] to total cholesterol (Appendix 2)

* Unadjusted

** Adjusted by age, gender, BMI and total activity

R^2^= corrected R^2^

β= influence factor on the dependent variable (standardized B)

p-values ≤0.05 are shown in bold

2 HEI-Flex score values: Score Points (SP) based on calculations with the Healthy Eating Index-flexible (HEI-Flex) according to [Bruns et al, 2022] with cut-off values (V) of: Vmax = 100 SP and Vmin = 0 SP; higher SP indicate higher diet quality

**B Linear regression models to examine associations of LDL levels with various food groups in the total study population**

| LDL | Model 1* | | | Model 2** | | |
| --- | --- | --- | --- | --- | --- | --- |
|  | *R^2^* | p-value | *β* | *R^2^* | p-value | *β* |
| Food Groups^1^ |  | | |  | | |
| Softdrinks | 0.099 | **0.001** | 0.328 | 0.209 | **0.006** | 0.273 |
| Vegetable | 0.064 | **0.008** | -0.273 | 0.197 | **0.012** | -0.254 |
| Fruit | 0.049 | **0.019** | -0.242 | 0.175 | **0.047** | -0.202 |
| Milk | 0.007 | 0.531 | 0.065 | 0.139 | 0.634 | 0.046 |
| Dairy | 0.074 | **0.005** | 0.289 | 0.165 | 0.091 | 0.172 |
| Plant-based milk alternatives | 0.011 | 0.935 | -0.009 | 0.137 | 0.844 | -0.020 |
| Plant-based dairy alternatives | 0.042 | **0.026** | -0.230 | 0.178 | 0.039 | -0.199 |
| Legumes | 0.073 | **0.005** | -0.289 | 0.188 | 0.021 | -0.236 |
| Nuts and Seeds | 0.018 | 0.104 | -0.169 | 0.144 | 0.395 | -0.089 |
| Sweets | 0.059 | **0.011** | 0.262 | 0.187 | **0.023** | 0.225 |
| Meat | 0.116 | **<0.001** | 0.354 | 0.193 | **0.015** | 0.248 |
| Processed meat products | 0.047 | **0.020** | 0.240 | 0.162 | 0.112 | 0.163 |
| Plant-based meat alternative products | 0.006 | 0.525 | -0.066 | 0.144 | 0.380 | -0.090 |
| Fish and fish products | 0.001 | 0.342 | 0.099 | 0.143 | 0.416 | 0.081 |
| Eggs | 0.008 | 0.618 | 0.052 | 0.138 | 0.741 | 0.032 |
| HEI-Flex^2^ | 0.081 | **0.003** | -0.301 | 0.188 | **0.020** | -0.236 |

1 Food Group selection base on significant Spearmans correlation coefficient rho ≤0.05 [J. Cohen, 1988] to LDL (Appendix 2)

* Unadjusted

** Adjusted by age, gender, BMI and total activity

R^2^= corrected R^2^

*β*= influence factor on the dependent variable (standardized regression coefficient B)

p-values ≤0.05 are shown in bold

2 HEI-Flex score values: Score Points (SP) based on calculations with the Healthy Eating Index-flexible (HEI-Flex) according to [Bruns et al, 2022] with cut-off values (V) of: Vmax = 100 SP and Vmin = 0 SP; higher SP indicate higher diet quality

**C Linear regression models to examine associations of MetS-score levels (based on BMI and waistline) with various food groups in the total study population**

|  | MetS-score (based on BMI) * | | | MetS-score (based on waistline) * | | |
| --- | --- | --- | --- | --- | --- | --- |
|  | *R^2^* | p-value | *β* | *R^2^* | p-value | *β* |
| Food Groups^1^ |  | | |  | | |
| Softdrinks | 0.003 | 0.698 | 0.040 | 0.002 | 0.641 | 0.049 |
| Vegetable | 0.067 | **0.013** | -0.263 | 0.095 | **0.002** | -0.337 |
| Fruit | 0.030 | 0.111 | -0.173 | 0.033 | 0.063 | -0.201 |
| Plant-based dairy | 0.018 | 0.230 | -0.125 | -.- | -.- | -.- |
| Nuts and Seeds | 0.016 | 0.251 | -0.122 | 0.024 | 0.104 | -0.173 |
| Sweets | 0.035 | 0.080 | 0.182 | 0.045 | **0.032** | 0.223 |
| Meat | 0.056 | **0.025** | 0.237 | 0.034 | 0.060 | 0.200 |
| Processed Meat | 0.080 | **0.007** | 0.286 | 0.078 | **0.005** | 0.293 |
| HEI-Flex^2^ | 0.101 | **0.002** | -0.327 | 0.102 | **0.001** | -0.340 |

1 Food Group selection base on significant Spearmans correlation coefficient rho ≤0.05 [J. Cohen, 1988] to both MetS-score levels (Appendix 2)

*Adjusted by total activity

R^2^= corrected R^2^

*β*= influence factor on the dependent variable (standardized regression coefficient B)

p-values ≤0.05 are shown in bold

2 HEI-Flex score values: Score Points (SP) based on calculations with the Healthy Eating Index-flexible (HEI-Flex) according to [Bruns et al, 2022] with cut-off values (V) of: Vmax = 100 SP and Vmin = 0 SP; higher SP indicate higher diet quality

**D Linear regression models to examine associations of PWV levels with various food groups in the total study population**

| PWV | Model 1* | | | | Model 2** | | |
| --- | --- | --- | --- | --- | --- | --- | --- |
|  | *R^2^* | p-value | *β* | *R^2^* | | p-value | *β* |
| Food Groups^1^ |  | | | |  | | |
| Meat | 0.062 | **0.009** | 0.268 | 0.213 | | **0.010** | 0.260 |
| Processed meat products | 0.060 | **0.010** | 0.265 | 0.199 | | **0.025** | 0.226 |
| Eggs | 0.005 | 0.230 | 0.125 | 0.160 | | 0.362 | 0.088 |

1 Food Group selection base on significant Spearmans correlation coefficient rho ≤0.05 [J. Cohen, 1988] to PWV (Appendix 2)

* unadjusted

** adjusted by age, gender, BMI and total activity

R^2^= corrected R^2^

*β*= influence factor on the dependent variable (standardized regression coefficient B)

p-values ≤0.05 are shown in bold
